# High quality draft genome of *Nakamurella lactea* type strain*,* a rock actinobacterium, and emended description of *Nakamurella lactea*

**DOI:** 10.1186/s40793-016-0216-0

**Published:** 2017-01-06

**Authors:** Imen Nouioui, Markus Göker, Lorena Carro, Maria del Carmen Montero-Calasanz, Manfred Rohde, Tanja Woyke, Nikos C. Kyrpides, Hans-Peter Klenk

**Affiliations:** 1 School of Biology, Newcastle University, Newcastle upon Tyne, NE1 7RY UK; 2 Leibniz Institute DSMZ, Inhoffenstr. 7 B, 38124 Braunschweig, Germany; 3 Central Facility for Microscopy, HZI—Helmholtz Centre for Infection Research, Braunschweig, Germany; 4 Department of Energy Joint Genome Institute, Walnut Creek, CA USA; 5 Department of Biological Sciences, Faculty of Science, King Abdulaziz University, Jeddah, Saudi Arabia

**Keywords:** *Frankineae*, Rare actinobacteria, *Nakamurellaceae*, Bioactive natural product, Next generation sequencing

## Abstract

*Nakamurella lactea* DLS-10^T^ *,* isolated from rock in Korea, is one of the four type strains of the genus *Nakamurella.* In this study, we describe the high quality draft genome of *N. lactea* DLS-10^T^ and its annotation. A summary of phenotypic data collected from previously published studies was also included. The genome of strain DLS-10^T^ presents a size of 5.82 Mpb, 5100 protein coding genes, and a C + G content of 68.9%. Based on the genome analysis, emended description of *N. lactea* in terms of G + C content was also proposed.

## Introduction

The genus *Nakamurella*, belong to the order Nakamurellales [1] and is one of the rare genera in the class *Actinobacteria* [2]. The genus *Nakamurella* is the sole and type genus of the family *Nakamurellaceae*, which replaced the family *Microsphaeraceae* [2] in 2004 [3]*.* The genus and family names were assigned in honour of the microbiologist Kazonuri Nakamura [4].

Only four species with validly published names, *Nakamurella multipartita* [3, 5]*,* *Nakamurella panacisegetis* [6, 7]*,* *Nakamurella flavida* [6–8], and *Nakamurella lactea* [6, 7, 9], have been described, and only the genome of *Nakamurella multipartita* has been published [10].

*N. lactea* was originally described as *Saxeibacter lacteus* [9], which was the type species of one of the three genera comprising in the family *Nakamurellaceae*. Then, in the light of the 16S rRNA gene and *rpoB* gene sequences similarities and chemotaxonomic features [6], the species was reclassified into the genus *Nakamurella*. *Nakamurella lactea* is represented by the type strain DLS-10^T^ (= DSM 19367 ^T^ = JCM 16024^T^ = KCTC 19285^T^).

The availability of the genome of one more species in the genus will provide vital baseline information for better understanding of the ecology of these rare actinobacteria and their potential as source of bioactive natural products. In the present study, we summarise the phenotypic, physiological and chemotaxonomic, features of *N. lactea* DLS-10^T^ together with the genomic data.

## Organism information

### Classification and features

**Fig. 1:**
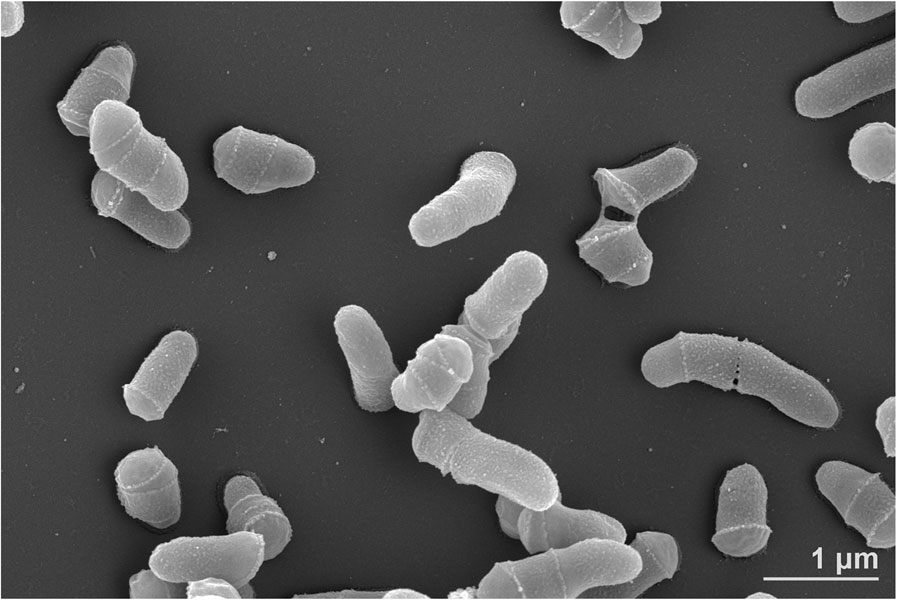
Scanning electron micrograph of *N. lactea* DLS-10^T^. The bacterium was grown on DSM medium 65 for 3 days at 28^∘^C

*N. lactea* DLS-10^T^ was isolated from a rock collected on the parasitic volcano Darangshi Oreum at 300 m above sea level in Jeju island, Republic of Korea (latitude 33.51, longitude 126.52) [9]. It has been shown by Lee et al. [9] and Kim et al. [4, 6] that its cells are aerobic, non-motile, non-spore and non-mycelium forming short rods with 0.4–0.7 μm and 0.9–1.0 μm of cell diameter and length, respectively (Fig. 1), producing cream-coloured colonies on TSA medium. A summary of the classification and general features of *N. lactea* strain DLS-10^T^ is presented in the Table 1. Additional phenotypic features can be found in Lee et al. and Kim et al. [6, 9].

**Table 1:** Classification and general features of *Nakamurella lactea* strain DLS-10^T^, according to the MIGS recommendations [36] as developed by [22]

MIGS ID	Property	Term	Evidence code^a^
	Classification	Domain *Bacteria*	TAS [39]
		Phylum *Actinobacteria*	TAS [40]
		Class *Actinobacteria*	TAS [2]
		Order *Nakamurellales*	TAS [1]
		Family *Nakamurellaceae*	TAS [41]
		Genus *Nakamurella*	TAS [3, 41]
		Species *Nakamurella lactea* Type strain DLS-10	TAS [6, 9]
	Gram stain	Positive	TAS [6, 9]
	Cell shape	Rod	TAS [6, 9]
	Motility	non-motile	TAS [6, 9]
	Sporulation	Non-sporulating	NAS [6, 9]
	Temperature range	4–37 °C	TAS [6, 9]
	Optimum temperature	25 °C	TAS [6, 9]
	pH range	5.1–9.1	TAS [6, 9]
	pH Optimum	6.0–7.0	
	Carbon source	L-Arabinose, myo-inositol and methyl α-D-mannoside, D-cellobiose, D-fructose, D-glucose, D-galactose, lactose, D-maltose, D-mannitol, D-mannose, L-rhamnose, salicin, sucrose and D-trehalose, D- turanose	TAS [6, 9]
MIGS-6	Habitat	Rock	TAS [9]
MIGS-6.3	Salinity	Up to 3% NaCl	TAS [6, 9]
MIGS-22	Oxygen requirement	Aerobic	TAS [9]
MIGS-15	Biotic relationship	free-living	TAS [9]
MIGS-14	Pathogenicity	non-pathogen	NAS
MIGS-4	Geographic location	Korea	TAS [9]
MIGS-5	Sample collection	Not reported	TAS []
MIGS-4.1	Latitude	33.51	TAS [9]
MIGS-4.2	Longitude	126.52	TAS [9]
MIGS-4.4	Altitude	300 m	TAS [9]

^a^Evidence codes are from of the Gene Ontology project [42]. *TAS* traceable author statement (i.e., a direct report exists in the literature)

Only four species isolated from soil (*N. panacisegetis* and *N. flavida*), rock (*N. lactea*) and sludge (*N. mutipartita*), respectively, are currently classified in the genus. Due to this limited number of the characterised species, the ecological diversity as well as the biotechnological potential of the members of the genus *Nakamurella* remain to be studied in depth.

**Fig. 2:**
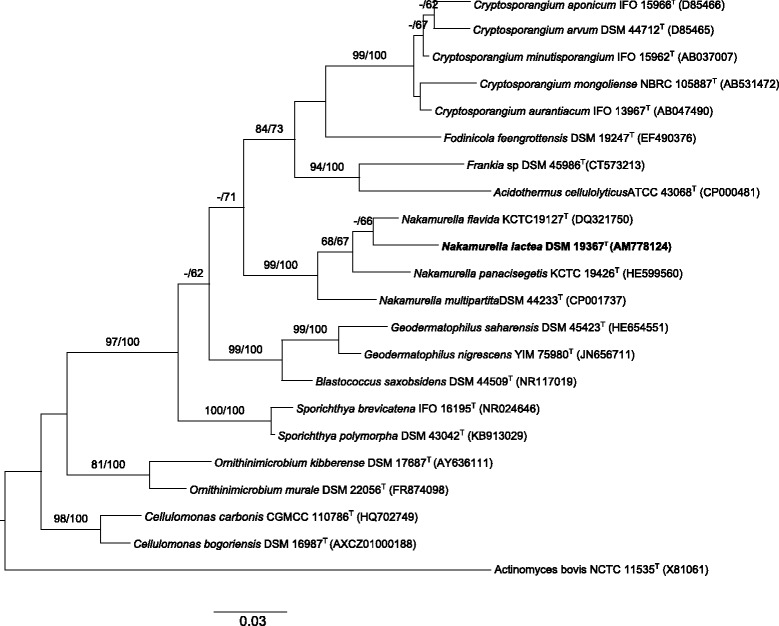
Maximum likelihood phylogenetic tree of *N. lactea* DLS-10^T^ and related type strains within the related families constructed under the GTR + GAMMA model and rooted using *Actinomyces bovis* NCTC 11535^T^ as outgroup. The branches are scaled in terms of the expected number of substitutions per site (see size bar). Support values from maximum-likelihood (left) and maximum-parsimony (right) bootstrapping are shown above the branches if equal to or larger than 60%

Phylogenies based on 16S rRNA gene sequences included in this manuscript were performed using the GGDC web server [11] implementation of the DSMZ phylogenomics pipeline [12]. The multiple alignment was created with MUSCLE [13] and maximum likelihood (ML) and maximum parsimony (MP) trees were inferred from it with RAxML [14] and TNT [15], respectively. For ML, rapid bootstrapping in conjunction with the autoMRE bootstopping criterion [16] and subsequent search for the best tree was used; for MP, 1000 bootstrapping replicates were used in conjunction with tree-bisection-and-reconnection branch swapping and ten random sequence addition replicates. This analysis shows the family *Nakamurellaceae* [4] as the sister group of the families *Cryptosporangiaceae*, *Sporichthyaceae*, and *Geodermatophilaceae*. The monophyly of the genus *Nakamurella* was supported by (close to) maximum bootstrap values under ML and MP (Fig. 2).

### Chemotaxonomic data (optional, Heading 3)

Glucose, mannose, ribose and rhamnose were detected as the whole-cell sugars [5]. The pattern of polar lipid contains diphosphatidylglycerol, phosphatidylethanolamine, phosphatidylinositol, aminophospholipid, five unidentified phosphoglycolipids, and one unidentified glycolipid [6].

The diagnostic peptidoglican is the meso-diaminopimelic acid. The major fatty acids are anteiso-C_15:0_, C_16:0_, iso-C_16:0_, and anteiso-C_17:0_ [9]. MK-8(H_4_) and MK-9(H_4_) are the predominant menaquinones but MK-7(H_4_) was also revealed in a low amount [6].

## Genome sequencing information

### Genome project history

*N. lactea* DLS-10^T^ (DSM 19367 ^T^) was selected for sequencing on the basis of its phylogenetic position [17, 18], and is part of Genomic Encyclopedia of Type Strains, Phase I: the one thousand microbial genomes project [19], a follow-up of the Genomic Encyclopedia of Bacteria and Archaea pilot project [20], which aims at increasing the sequencing coverage of key reference microbial genomes and to generate a large genomic basis for the discovery of genes encoding novel enzymes [21]. KMG-I is the first of the production phases of the “Genomic Encyclopedia of Bacteria and Archaea: sequencing a myriad of type strains” initiative [22] and a Genomic Standards Consortium project [23]. The project and the genome sequence are deposited in the Genome OnLine Database [24] and Genbank under the accession number AUFT00000000.1. In Table 2, we summarize genome sequence project.

**Table 2:** Project information

MIGS ID	Property	Term
MIGS 31	Finishing quality	Level 1: Standard Draft
MIGS-28	Libraries used	NOHX
MIGS 29	Sequencing platforms	Illumina, Illumina HiSeq 2000
MIGS 31.2	Fold coverage	NA
MIGS 30	Assemblers	Allpaths/Velvet
MIGS 32	Gene calling method	Prodigal 2.5
	Locus Tag	K340
	Genbank ID	AUFT00000000.1
	GenBank Date of Release	2013-06-03
	GOLD ID	Gi11889
	BIOPROJECT	PRJNA195807
MIGS 13	Source Material Identifier	DSM 19367^T^
	Project relevance	GEBA-KMG, Tree of Life

### Growth conditions and genomic DNA preparation

A *N. lactea* DLS-10^T^ culture was prepared in DSM medium 65 [25] at 28 °C. Genomic DNA was extracted using MasterPure™ Gram Positive DNA Purification Kit (Epicentre MGP04100) following the standard protocol provided by the manufacturer but modified by the incubation on ice overnight on a shaker, the use of additional 1 μl proteinase K, and the addition of 7.5 units achromopeptidase, 7.5 μg/μl lysostaphine, 1050.0 units lysozyme, and 7.5 units mutanolysine. DNA is available from DSMZ through the DNA Bank Network [26].

### Genome sequencing and assembly

The draft genome of *N. lactea* DLS-10^T^ was generated at the DOE Joint genome Institute (JGI) using the Illumina technology [27]. An Illumina standard shotgun library was constructed and sequenced using the Illumina HiSeq 2000 platform, which generated 13,910,936 reads totalling 2,086.6 Mb. All general aspects of library construction and sequencing performed at the JGI can be found at http://www.jgi.doe.gov. All raw Illumina sequence data was passed through DUK, a filtering program developed at JGI, which removes known Illumina sequencing and library preparation artefacts (unpublished results). Following steps were then performed for assembly: (1) filtered Illumina reads were assembled using Velvet (version 1.1.04) [28], (2) 1–3 kb simulated paired end reads were created from Velvet contigs using wgsim (https://github.com/lh3/wgsim), (3) Illumina reads were assembled with simulated read pairs using Allpaths–LG (version r42328) [29]. Parameters for assembly steps were: 1) Velvet (velveth:63 –shortPaired and velvetg: −very clean yes –exportFiltered yes –min contig lgth 500 –scaffolding no–cov cutoff 10) 2) wgsim (−e 0 –1 100 –2 100 –r 0 –R 0 –X 0) 3) Allpaths–LG (PrepareAllpathsInputs:PHRED 64 = 1 PLOIDY = 1 FRAG COVERAGE = 125 JUMP COVERAGE = 25 LONG JUMP COV = 50, RunAllpathsLG: THREADS = 8 RUN = std shredpairs TARGETS = standard VAPI WARN ONLY = True OVERWRITE = True). The final draft assembly contained 31 contigs in 27 scaffolds. The total size of the genome is 5.8 Mb and the final assembly is based on 712.8 Mb of Illumina data, which provides an average 122.5X coverage of the genome.

### Genome annotation

The complete genome sequence was annotated using the JGI Prokaryotic Automatic Annotation Pipeline [30] with additional manual review using the Integrated Microbial Genomes - Expert Review (IMG-ER) platform [31]. The predicted CDSs were translated and used to search the National Center for Biotechnology Information (NCBI) non redundant database, UniProt, TIGRFam, Pfam, KEGG, COG, and InterPro databases. The tRNAScanSE tool [32] was used to find tRNA genes, whereas ribosomal RNA genes were found by searches against models of the ribosomal RNA genes built from SILVA [33]. Other non–coding RNAs such as the RNA components of the protein secretion complex and the RNase P were identified by searching the genome for the corresponding Rfam profiles using INFERNAL [34]. Additional gene prediction analysis and manual functional annotation was performed within the Integrated Microbial Genomes (IMG) platform [35, 36] developed by the Joint Genome Institute, Walnut Creek, CA, USA [37].

## Genome properties

The 5820860 bp of genome size of *N. lactea* DLS-10^T^ presents 5100 protein-coding genes, 3 rRNA genes (5S, 16S, 23S RNA) and 59 tRNA genes. A G + C content of 68.9% was calculated. More genome details are listed in Tables 3 and 4.

**Table 3:** Genome statistics

Attribute	Value	% of Total
Genome size (bp)	5820860	100.00
DNA coding (bp)	5332245	91.61
DNA G + C (bp)	4011790	68.92
DNA scaffolds	27	100.00
Total genes	5169	100.00
Protein coding genes	5100	98.67
RNA genes	69	1.33
Pseudo genes	231	
Genes in internal clusters	588	11.38
Genes with function prediction	4048	78.31
Genes assigned to COGs	3321	64.25
Genes with Pfam domains	4211	81.47
Genes with signal peptides	432	8.36
Genes with transmembrane helices	1206	23.33
CRISPR repeats	1

**Table 4:** Number of genes associated with general COG functional categories

Code	Value	%	Description
J	198	5.07	Translation, ribosomal structure and biogenesis
A	1	0.03	RNA processing and modification
K	392	10.04	Transcription
L	122	3.12	Replication, recombination and repair
B	1	0.03	Chromatin structure and dynamics
D	25	0.64	Cell cycle control, Cell division, chromosome partitioning
V	94	2.41	Defence mechanisms
T	137	3.51	Signal transduction mechanisms
M	144	3.69	Cell wall/membrane biogenesis
N	10	0.26	Cell motility
U	23	0.59	Intracellular trafficking and secretion
O	121	3.1	Posttranslational modification, protein turnover, chaperones
C	210	5.38	Energy production and conversion
G	648	12.31	Carbohydrate transport and metabolism
E	459	11.75	Amino acid transport and metabolism
F	91	2.33	Nucleotide transport and metabolism
H	219	5.61	Coenzyme transport and metabolism
I	255	6.53	Lipid transport and metabolism
P	244	6.25	Inorganic ion transport and metabolism
Q	154	3.94	Secondary metabolites biosynthesis, transport and catabolism
R	443	11.34	General function prediction only
S	158	44.05	Function unknown
-	1848	35.75	Not in COGs

## Conclusion

The genome of *N. lactea* will be used to study, for the first time, its potential as bioactive natural products source and the correlation between the rare soil bacteria and their habitat. According to [38], the within-species deviation in genomic G + C content is at most 1%. The range of 70.4–74.3% given in by Kim et al. [6] is thus too broad and too deviating from the 68.9% calculated in the genome sequence, much like the value 74.3% provided by Lee et al. [9]. This calls for an emendation of the species description [38].

## Emended description of *Nakamurella lactea* (Lee et al. [9]) Kim et al. [6]

The properties are as given in the species description by Kim et al. [6] with the following emendation. Based on the genomic data the G + C content is 68.9%.
